# Poly‐GA immunohistochemistry is a reliable tool for detecting *C9orf72* hexanucleotide repeat expansions

**DOI:** 10.1111/bpa.13216

**Published:** 2023-10-10

**Authors:** Jordan Carroll, Heather McCann, Glenda M. Halliday, John B. Kwok, Carol Dobson‐Stone, Claire E. Shepherd

**Affiliations:** ^1^ Neuroscience Research Australia Randwick New South Wales Australia; ^2^ Faculty of Medicine and Health School of Medical Sciences, University of Sydney Brain and Mind Centre Camperdown New South Wales Australia; ^3^ School of Medical Sciences University of New South Wales Kensington New South Wales Australia

**Keywords:** *C9orf72*, poly‐GA, TDP‐43

## Abstract

Poly‐GA immunohistochemistry (A) on formalin fixed paraffin embedded cerebellum sections shows a similar distribution to p62 antibody (B) and reliably identifies neuronal cytoplasmic inclusions and neurites in cases with known *C9orf72* repeat expansion. This is useful in the research setting where genetic testing has not been performed in life or suitable tissue is not avilable post‐mortem.

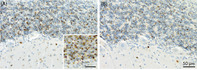

## ETHICS STATEMENT

The Sydney Brain Bank has ethical approval from the University of New South Wales, Australia, to collect, characterise, store and distribute human brain tissue for the purpose of medical research. The recruiting clinical brain donor programs hold ethical approvals from the University of Sydney and Macquarie University.

An intronic hexanucleotide repeat expansion (GGGGCC) in the gene *chromosome 9 open reading frame 72* (*C9orf72*) is one of the most frequent genetic alterations causative for neurodegenerative disease and the most common genetic cause of frontotemporal lobar degeneration (FTLD) and motor neuron disease (MND) [1]. As such, genetic testing for repeat expansions is increasingly recommended for both FTLD and MND patients [2]. The neuropathology of individuals with a *C9orf72* intronic repeat expansion is characterised by the abnormal accumulation of TAR DNA binding protein 43 (TDP‐43) [3]. In addition, *C9orf72*‐related MND/FTLD cases also accumulate dipeptide repeat proteins (DPR), including poly Gly‐Pro (poly‐GP), poly Gly‐Arg (poly‐GR), poly Ala‐Pro (poly‐AP), and poly Pro‐Arg (poly‐PR) [4]. Poly‐GA is the dominant form that accumulates in neuronal cytoplasmic inclusions (NCIs) [5], and these NCIs are most frequently found in the cerebellum, hippocampus, and neocortex in FTLD‐TDP and MND‐TDP [4]. We have used an antibody to detect the poly‐GA DPR as it has been demonstrated to be the most prevalent in a series of both FTLD‐TDP and MND‐TDP cases harbouring the *C9orf72* expansion [4], as well as the main aggregating species of DPR in FTLD‐UPS cases with *C9orf72* repeat expansions [6].

We have screened all cases neuropathologically characterised with FTLD‐TDP (n = 63) and MND‐TDP (*n* = 64) (Table 1) at the Sydney Brain Bank according to current neuropathological diagnostic criteria [7, 8] to determine the reliability of poly‐GA immunohistochemistry for the detection of the *C9orf72* repeat expansion in post‐mortem brain tissue. Cases for this study were recruited through prospective ageing and neurodegeneration brain donor programmes. The Sydney Brain Bank has ethical approval from the University of New South Wales, Australia, to collect, characterise, store and distribute human brain tissue for the purpose of medical research. The recruiting clinical brain donor programmes hold ethical approvals from the University of Sydney and Macquarie University. Of the 127 neuropathologically characterised cases with post‐mortem brain tissue samples, 114 had been tested for the repeat expansion in *C9orf72* through repeat‐primed PCR amplification and capillary electrophoresis using genomic DNA from peripheral blood lymphocytes or frozen brain tissue (where blood was not available) [3]. Samples were scored as expansion‐positive (pathogenic) if they harboured >30 repeats (*n* = 26). Thirteen cases had not undergone genetic testing at the time this study was carried out (Table 1).

**TABLE 1:** Summary of poly‐GA immunohistochemistry on cerebellum tissue compared to genetic testing results, average age and post‐mortem delay (PMD) shown as mean ± standard deviation.

Neuropathological diagnosis	N	Age (years)	PMD (hours)	*C9orf72* repeat expansion (negative/positive)	Poly‐GA (negative/positive)
FTLD‐TDP	63	69 ± 9	25 ± 18	(35/18)	(43/20)
MND	64	64 ± 11	28 ± 15	(52/9)	(52/12)
Total	127			114	127

**FIGURE 1:**
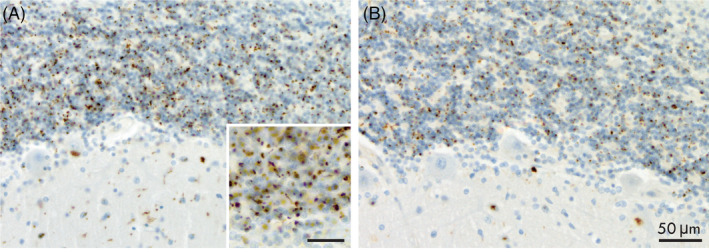
Immunostaining showing similar distribution of NCIs and neurites in cerebellar cortex and granule cell layers using poly‐GA antibody (A) and p62 antibody (B), 200× magnification. Staining was robust in all positive cases allowing 100% inter‐rater reliability. Inset in (A) shows poly‐GA NCIs and neurites at 400× magnification.

Donated whole brains were fixed in 15% neutral buffered formalin for 2 weeks then sliced into 3 mm coronal slices with standard tissue blocks taken for standard neuropathological analysis, as previously described [9]. Additional paraffin‐embedded cerebellar sections were cut on a rotary microtome at 10 μm thickness and poly‐GA immunostaining performed using a commercially available antibody (MABN889 anti‐poly‐GA antibody, 1 mg/mL, Millipore) and a BenchMark GX autostainer with Optiview Detection kit (cat# 760–700 Roche Diagnostics) and haematoxylin counterstain. The cerebellar cortex was selected as NCIs are reported to be frequently seen in this region [4] as well as having highest levels of insoluble protein for both FTLD‐TDP and MND‐TDP [6] and our experience concurs with this observation. Slides were assessed blind to genetic testing results by two investigators (JC and HM) at 200× magnification using a Zeiss AxioLab microscope and the presence or absence of poly‐GA immunopositive NCIs and neurites was noted. Poly‐GA pathology was present in both the molecular and granule cell layers (Figure 1). Inter‐rater agreement between two investigators was 100% as there were no instances of equivocal staining and results were robust in all positive cases. Poly‐GA staining was seen in the same distribution as p62 immunopositivity (Figure 1) and, in agreement with another study, we found no staining effects associated with variable post‐mortem delay or prolonged formalin fixation [4].

Poly‐GA antibody positivity was then correlated to the presence of *C9orf72* repeat expansion status. 100% of cases with known *C9orf72* repeat expansion were identified with the poly‐GA antibody (*n* = 26). A further five cases were found to be poly‐GA immunopositive. For 2/5 cases, either blood or fresh frozen brain tissue was available for genomic DNA extraction and both were confirmed positive for the *C9orf72* repeat expansion. Of the other, three cases that had no blood or brain tissue available for screening, one had a sibling with *C9orf72* repeat expansion and the other two had no known genetic testing results or family history.

This study was performed to test the hypothesis that poly‐GA immunohistochemistry using a commercially available antibody is a reliable and sensitive method of detecting *C9orf72* repeat expansions in post‐mortem FTLD‐TDP and MND‐TDP brain tissues in a research setting. Neuropathological detection of these proteins is economical and sensitive compared to genetic testing, which is currently considered to be the gold standard for detecting *C9orf72* repeat expansions [2, 10]. While repeat‐primed PCR for the *C9orf72* repeat expansion is cheaper and faster, this method of testing has been shown to have limitations in sensitivity, specificity and inter‐laboratory interpretation of results, and has limited capacity to accurately identify large repeat sizes [2]. Interestingly, two of our cases that were negative for poly‐GA staining were identified with intermediate *C9orf72* repeat numbers of 22 and 26. Although both cases displayed clinical disease presentation (one FTD and one MND) and these phenotypes were pathologically confirmed as FTLD‐TDP and MND‐TDP, an intermediate repeat length was not sufficient to cause aberrant poly‐GA aggregation. Given the small number of intermediate repeat cases in this study and uncertainty around the clinical effects of repeats in this intermediate range [2] further investigation of the interactions between *C9orf72* repeat size, clinical phenotype, and poly‐GA pathology is required to gain more insight into this issue. Strengths of this study include a reasonably large FTLD‐TDP and MND‐TDP cohorts with clear and standardised neuropathological diagnoses, *C9orf72* repeat expansion screening results on the majority of cases and the systematic use of an autostainer for consistent immunohistochemistry results. The main weakness of this study is that we do not have *C9orf72* repeat expansion screening results for 100% of cases.

In summary, poly‐GA immunohistochemistry of post‐mortem human brain tissue serves as a useful tool to accurately identify the presence of toxic DPR pathology because of pathogenic expansions in the *C9orf72* gene. This is especially useful where genetic testing cannot be performed in life or where suitable tissue is not available post‐mortem.

## AUTHOR CONTRIBUTIONS

Jordan Carroll, Claire Shepherd and Heather McCann designed the study. John Kwok, Carol Dobson‐Stone and Glenda Halliday provided genetic testing results and expertise. Jordan Carroll and Heather McCann performed assessment of poly‐GA pathology. All authors were involved in editing and approving the manuscript.

## FUNDING INFORMATION

Glenda M. Halliday was supported by an NHMRC Senior Leadership Fellowship (1176607). Carol Dobson‐Stone was supported by the NHMRC Boosting Dementia Leadership Fellowship (1138223).

## CONFLICT OF INTEREST STATEMENT

We have none to declare.

## Data Availability

The data that support the findings of this study are available on reasonable request from the corresponding author. The data are not publicly available due to privacy or ethical restrictions.

## References

[1] DeJesus‐Hernandez M , Mackenzie IR , Boeve BF , Boxer AL , Baker M , Rutherford NJ , et al. Expanded GGGGCC hexanucleotide repeat in noncoding region of C9ORF72 causes chromosome 9p‐linked FTD and ALS. Neuron. 2011;72(2):245–256. 21944778 10.1016/j.neuron.2011.09.011 PMC3202986

[2] Crook A , McEwen A , Fifita JA , Zhang K , Kwok JB , Halliday G , et al. The C9orf72 hexanucleotide repeat expansion presents a challenge for testing laboratories and genetic counseling. Amyotroph Lateral Scler Frontotemporal Degener. 2019;20(5–6):310–316. 30907153 10.1080/21678421.2019.1588904

[3] Renton AE , Majounie E , Waite A , Simon‐Sanchez J , Rollinson S , Gibbs JR , et al. A hexanucleotide repeat expansion in C9ORF72 is the cause of chromosome 9p21‐linked ALS‐FTD. Neuron. 2011;72(2):257–268. 21944779 10.1016/j.neuron.2011.09.010 PMC3200438

[4] Mann DM , Rollinson S , Robinson A , Bennion Callister J , Thompson JC , Snowden JS , et al. Dipeptide repeat proteins are present in the p62 positive inclusions in patients with frontotemporal lobar degeneration and motor neurone disease associated with expansions in C9ORF72. Acta Neuropathol Commun. 2013;1:68. 24252525 10.1186/2051-5960-1-68 PMC3893586

[5] Mackenzie IR , Frick P , Grasser FA , Gendron TF , Petrucelli L , Cashman NR , et al. Quantitative analysis and clinico‐pathological correlations of different dipeptide repeat protein pathologies in C9ORF72 mutation carriers. Acta Neuropathol. 2015;130(6):845–861. 26374446 10.1007/s00401-015-1476-2

[6] Mori K , Weng SM , Arzberger T , May S , Rentzsch K , Kremmer E , et al. The C9orf72 GGGGCC repeat is translated into aggregating dipeptide‐repeat proteins in FTLD/ALS. Science. 2013;339(6125):1335–1338. 23393093 10.1126/science.1232927

[7] Brettschneider J , del Tredici K , Toledo JB , Robinson JL , Irwin DJ , Grossman M , et al. Stages of pTDP‐43 pathology in amyotrophic lateral sclerosis. Ann Neurol. 2013;74(1):20–38. 23686809 10.1002/ana.23937 PMC3785076

[8] Mackenzie IR , Neumann M . Reappraisal of TDP‐43 pathology in FTLD‐U subtypes. Acta Neuropathol. 2017;134(1):79–96. 28466142 10.1007/s00401-017-1716-8

[9] McCann H , Bahar AY , Burkhardt K , Gardner AJ , Halliday GM , Iverson GL , et al. Prevalence of chronic traumatic encephalopathy in the Sydney brain Bank. Brain Commun. 2022;4(4):fcac189. 35950093 10.1093/braincomms/fcac189 PMC9356727

[10] Akimoto C , Volk AE , van Blitterswijk M , Van den Broeck M , Leblond CS , Lumbroso S , et al. A blinded international study on the reliability of genetic testing for GGGGCC‐repeat expansions in C9orf72 reveals marked differences in results among 14 laboratories. J Med Genet. 2014;51(6):419–424. 24706941 10.1136/jmedgenet-2014-102360 PMC4033024

